# Severe Reproductive Disorders After Abdominal Fat Necrosis in Dairy Cattle

**DOI:** 10.3390/life15081182

**Published:** 2025-07-25

**Authors:** Vasilică Gotu, Sorin Aurelian Pașca, Ștefan Gregore Ciornei, Dragoș Constantin Anița, Daniela Porea, Geta Pavel, Răzvan Nicolae Mălăncuș, Gheorghe Savuța, Mariana Ioniță, Gheorghe Solcan, Ioan Liviu Mitrea

**Affiliations:** 1 Faculty of Veterinary Medicine, University of Agronomical Sciences and Veterinary Medicine Bucharest, 105 Independency Spl., 050097 Bucharest, Romania; gotuvasilica@yahoo.com (V.G.); mariana.ionita@fmvb.usamv.ro (M.I.); ioan-liviu.mitrea@fmvb.usamv.ro (I.L.M.); 2 Faculty of Veterinary Medicine, Iași University of Life Sciences, 8 M. Sadoveanu Alley, 700489 Iași, Romania; sorin.pasca@iuls.ro (S.A.P.); dragos.anita@iuls.ro (D.C.A.); daniela.porea@yahoo.com (D.P.); geta.pavel@iuls.ro (G.P.); razvan.malancus@iuls.ro (R.N.M.); gheorghe.savuta@iuls.ro (G.S.)

**Keywords:** dairy cows, infertility, high fat diet, obesity, genital lipogranulomas, inflammation

## Abstract

Abdominal fat necrosis is a dystrophic–necrotic process that is relatively common in dairy cows. It is determined by productive strain (excess fat in the diet), negative energy balance after calving, a lack of physical activity, vitamin E and selenium deficiency, etc. Lipomatous masses are predominantly located in the omentum and mesentery in cattle, potentially causing intestinal obstruction. We report on an outbreak of abdominal fat necrosis that affected 135 of 220 cows and heifers (61.36%); this involved massive fat accumulation in the uterine and salpingian ligaments and severe reproductive disorders (reducing fertility to 20% in cows and 10% in heifers) caused by a hyperenergetic diet (supplementation with saturated fats). A transrectal ultrasound examination of the genital apparatus—both in heifers and in cows in the puerperium—revealed a diffuse pathological hyperechogenicity of the cervical folds, suggesting lipid infiltration, proliferation of the endocervical folds and hyperechogenic lipogranulomas located paracervically or in the uterine ligaments. An ultrasound examination of the ovaries showed the presence of parasalpingial lipogranulomas on the mesovarium, with a uniformly pixelated greasy appearance, that altered the topography of the salpinx, leading to the impossibility of oocyte retrieval. At the histopathological examination, in addition to the necrosis of adipocytes and the subacute–chronic inflammation of the abdominal and retroperitoneal adipose tissue, lipid infiltration of the uterine walls was also observed in the uterine ligaments and lymph nodes. Additionally, lipid infiltration was observed in the wall of the uterine artery. All muscular-type branches of the ovarian artery exhibited subendothelial (subintimal) amyloid deposits, severely reducing their lumen and leading to ischaemia. Amyloidosis was secondary to the systemic inflammatory process triggered by lipid deposition and necrosis. Fertility returned to normal 45–60 days after the exclusion of fat supplements from the diet and their replacement with a vitamin–mineral supplement rich in antioxidants.

## 1. Introduction

Abdominal fat necrosis is a dystrophic–necrotic process that is relatively common in cattle, as well as in horses, pigs, etc. [1,2,3], and associated with steatitis (adipose tissue inflammation). Its clinical expression is usually due to intra-abdominal fat inflammation. It is also known as lipogranulomatosis, granulomatous steatitis, lipomatosis or lipofibromatosis (the latter two names being controversial [3]), or a variant of generalised steatitis [2]. The colloquial name is ‘yellow fat disease’ because of the colour of the affected tissues—a result of the accumulation of lipofuscin and fat oxidation products [3,4]. It is characterised by the appearance of hard necrotic fat masses in the peritoneal cavity of adult cattle, especially in the Jersey and Aberdeen Angus breeds, which can be confused with pregnancy or cause intestinal obstruction. The latter develops slowly, leading to moderate abdominal colic and terminal intestinal occlusion (the passage of small amounts of faeces). As the manifestations are initially subtle, most cases are detected during routine transrectal examinations for diagnosing pregnancy [2,5]. Lipomatous masses are located in the omentum and mesentery in cattle and more diffusely in other regions in sheep and goats. The composition of fat deposits is almost identical to that of healthy cows [2].

The disease is usually sporadic, but a prevalence of up to 67% has been described in steers grazing tall fescue, in which serum cholesterol was abnormally low [2]. Although the aetiopathogenesis is apparently not fully understood, there is a direct relationship with intensive production, as well as with excess fat in the diet and vitamin E and selenium deficiency. In goats, it has also been reported in fescue mycotoxicosis [6], and a hereditary predisposition is also suspected [2]. Stuedemann et al. [7] reported a prevalence of 60% in cows receiving high-nitrogen-fertilised fescue. The incidence rate increases with age, peaking at 7 years of age. It has been shown that the excessive accumulation of abdominal adipose tissue can predispose cattle to liponecrosis [8,9]. An unusual form of the disease, with numerous lesions in the subcutaneous areas, has been reported in Holstein Friesian cattle. This form is considered to be hereditary and there is no treatment [2]. On the other hand, high concentrations of plasma non-esterified fatty acids (NEFAs), a direct measure of lipolysis, are considered a risk factor for abomasal displacement (AD) and other associated clinical manifestations.

The most critical period in dairy farming is the transition period (the period 3 weeks before and 3 weeks after calving), when there is a rapidly increased need for the substances necessary for foetus development and milk synthesis. Since milk production rapidly increases from zero to the quantities needed for calf nutrition, the adjustment must be quick, leading to a mismatch between needs and adaptability. Due to these reasons, metabolic disorders often occur at the beginning of lactation [10].

In monogastric animals, uncontrolled lipolysis is frequently associated with macrophage infiltration of adipose tissue. In dairy cows, recent studies report the infiltration of specific fat deposits during the first week of lactation [11].

In cattle, the disease is characterised by the inflammation and necrosis of the fat in the abdominal cavity. It can be subclinical, with lesions detected during transrectal examination for pregnancy diagnosis or for other reasons [2]. The clinical signs of the disease are usually attributed to compressive lesions (compression of the rectum) or intestinal obstruction as a result of intestinal constriction by mesenteric fat accumulations and intestinal lumen occlusion [1,2]. Its clinical expression can vary from subclinical to a loss of appetite, decreased milk production, persistent diarrhoea, mild recurrent colic, acute colic, dystocia, urinary retention and coprostasis. Decline of fertility in dairy cows (30% lower) is frequently suggested to arise from the occurrence of a more negative energy balance and/or the concomitant increased accumulation of triacylglycerol in the liver, as well as other lipid disorders [12,13]. The masses can be detected during rectal examination or laparotomy [2]. The lesions consist of firm masses present in any portion of the omental, mesenteric or retroperitoneal fat, or as mobile structures floating freely in the abdomen. The masses vary from small nodules to large, solid, irregularly shaped formations. Ultrasonography can be used as an effective noninvasive tool to lead to a diagnosis of potential abdominal fat necrosis in cattle [14]. Lesions in cattle are rarely pedunculated [11]. Pathological fat deposition around the stomach, manifesting as persistent vomiting, has also been reported [15].

We report on an outbreak of diffuse steatitis and lipogranulomatosis in Holstein Friesian dairy cows, with massive lipogranuloma accumulation both in the abdominal fat and genital tract (in the uterus, paracervical, uterine and salpingian ligaments) leading to severe reproductive disorders as a result of a high-energy diet (productive forcing). To the authors’ knowledge, until now, the main pathological problems related to abdominal fat necrosis have focussed on digestive disorders, and therefore, our main goal was to demonstrate the reproductive disorders caused by development of lipogranulomas in segments of the genital system.

## 2. Materials and Methods

Investigations were carried out under production conditions at a dairy farm with Holstein Friesian cows, following a dramatic decline in reproductive parameters. A detailed clinical examination, gynaecological examination and transrectal ultrasound were performed using a Honda Electronics HS-1600V ultrasound scanner (Honda Electronics Co., Toyohashi, Japan), equipped with a 5–7.5 MHz Doppler transducer.

Haematological, biochemical and microbiological tests were performed on cows with reproductive disorders (repeated insemination, infertility and changes in the genital tract on gynaecological or ultrasound examination). Blood samples for the haematological and biochemical tests were collected from 20 cows from the jugular vein during the morning (8.00–10.00 a.m.) in the farm before transportation to the slaughterhouse. A complete blood count was performed using Abaxis VetScan HM5 (Zoetis Services, Parsippany, NJ, USA). The blood biochemistry test was performed using Abaxis VetScan V2 (Zoetis Services, Parsippany, NJ, USA)—VetScan Comprehensive Diagnostic Profile. The following parameters of interest for liver pathology and energy metabolism were determined: aspartate aminotransferase (AST), alanine aminotransferase (ALT), alkaline phosphatase (ALP), triglycerides (TGs), gamma-glutamyltransferase (GGT), serum cholesterol (CHOL), total bilirubin (TBIL), total proteins, serum albumin (ALB), blood glucose, urea, creatinine and the main electrolytes, calcium (Ca), phosphorus (P), magnesium (Mg), sodium (Na) and potassium (K).

In 24 cows slaughtered out of necessity, a gross and histopathological examination of the genital tract and affected internal organs was performed. Briefly, after the cows were slaughtered, all samples taken from the uterus, uterine horns, uterine ligaments and local lymph nodes, kidneys, pancreas and blood vessel walls (aorta, uterine artery) were fixed in 10% buffered formalin for 48 h and then embedded in paraffin with a Leica TP1020 tissue processor (Leica Microsystems GmbH, Wetzlar, Germany). Sections of 5 μm thickness were obtained with a Microtome SLEE CUT 6062 (SLEE Medical GmbH, Nieder-Olm, Germany), deparaffinised and stained using the Masson trichrome techniques. Qualitative histology was performed from stained sections using a Leica DM 1000 light microscope (Leica Microsystems GmbH, Germany) with an attached Leica ICC50 HD digital camera (Leica Microsystems GmbH, Germany). The microphotographs were created with Leica Application Suit Software (LAS) version 4.2.

A microbiological examination was performed to rule out bacterial infections. In order to rule out *Mycobacterium* spp., smears were taken from nodular lesions (lipogranulomas from all locations—mesenteric fat, abdominal fat, genital tract, including parauterine lymph nodes) and stained using the Ziehl–Neelsen reference method, and were then examined using an OLYMPUS CX31 microscope (Olympus Europe, Hamburg, Germany). The tissue samples were decontaminated via the double incubation method. We used hexadecylpyridinium chloride (HPC) as the decontamination agent, according to the working protocol for mycobacteria described in the Manual of Diagnostic Tests and Vaccines for Terrestrial Animals [16]. Lowenstein–Jensen commercial and Middlebrook 7H9 media were used for cultivation. Due to the long incubation time required for mycobacterial growth on culture media, until 21 days [17], molecular diagnostic methods—polymerase chain reaction (PCR)—were used for rapid identification in 5 cattle with nodular uterine lesions, using the MJ Mini™ thermocycler, BIO-RAD. A commercial extraction kit, NucleoSpin^®®^ Tissue MACHEREY-NAGEL GmbH & Co. KG (Düren, Germany), was used to extract total DNA from tissues following the protocol and instructions specified by the manufacturer. A sample of bird liver with lesions specific to avian tuberculosis, stored in the bacteriology laboratory’s sample bank, was used to validate the extraction. The use of the 16S rRNA target ensured rapid mycobacterial identification. Its use has contributed significantly to the discovery of new species within the Mycobacterium genus. The RpoB gene is also used for species identification and has proven to be very useful for identifying isolates of veterinary origin that mostly belong to the MAC complex.

To perform the PCR, nine samples were used, of which one was a negative control; five samples were extracts from bovine tissues, one sample was an extract from chicken liver and two samples were used as positive controls. The positive controls used were two extracts previously identified as *Mycobacterium septicum/porcinum* (M+1) and MAC species (M+2). The following primers were used to perform PCR: Sense primer 5′ ACC AAC GAT GGT GTG TCC AT 3′ and Antisense primer 3′ CTT GTC GAA CCG CAT ACC CT 5′. These primers amplified a fragment of 439 base pairs [17].

The EU Commission’s 2007 recommendation, the European Parliament’s Directive 2010/63/EU and the Council’s 22 September 2010 standards for the housing, care and protection of animals used for experiments and other scientific reasons were all followed in the maintenance and treatment of the animals. Our study was conducted with the approval of the Ethics Commission of the Faculty of Veterinary Medicine, Iași University of Life Sciences (nr. 1722/5.09.2022).

## 3. Results

The outbreak of abdominal fat necrosis, which affected 135 of 220 cows and heifers (61.36%), progressively evolved three months after a new saturated fat-based ingredient was introduced into their feed (Table 1, Formula 1). The situation improved 45–60 days after replacing the fat supplement with a multivitamin and trace elements (Table 1, Formula 2)

A general clinical examination found that the cows were in good to very good condition and had high genetic potential, with an average daily milk production of 34 L. The animals’ height was above the average for the H.F. breed. The average body score on the farm was 3–4 (heifers 4, cows 3). Frequent foot disorders (laminitis, abscesses) were observed. Feeding was differentiated only by age and physiological status, not by productive performance; however, with good quality feed and nutritional supplements, the ration was hyperenergetic for lactating cows (Table 1). Heifers received the same ration as weaned cows.

In the cows during the puerperal period, transrectal examination revealed nodular formations of various sizes (from 0.5–1 cm to 8–12 cm) and shapes in the pelvic–genital area, mainly in the uterus. Both large single and multiple formations with a pearly nodular appearance, located near the cervix, particularly affecting its serosa and sometimes extending towards the broad ligaments of the uterus, were identified. In cows in late puerperium, the uterus sometimes resembled a homogeneous block that included the cervix, broad ligaments and uterine horns. This looked like pregnancy at transrectal examination. The consistency of the identified formations was increased, and they appeared to be hard, elastic and with a rubbery appearance, with no increased sensitivity in the implantation area. The uteri of the cows in puerperium were physiologically involuted, showing signs of resumed functionality. In cows with such formations, the topography of the cervix was changed, the diameter was large (>10 cm) and artificial insemination could not be performed correctly. In these cows, gynaecological examination became almost impossible, along with all the procedures that depend on it (detection of uterine segments, examination of ovarian function, artificial insemination, diagnosis of pregnancy). However, in cows examined in early and late puerperium, as well as in those calved more than 90 days before, ovarian activity resumed and the cows exhibited spontaneous oestrus phases according to breed and productivity level. Following artificial insemination (with spontaneous or induced oestrus), the fertility rate dropped dramatically 3 months after the introduction of the saturated fat supplement into the feed, from 40–60% to 20% in cows and 10% in heifers.

In heifers, the genital tract as a whole was physiologically developed and functional. Ovarian activity was normal and oestrus occurred regularly and clinically well manifested. Most heifers ready for reproduction (14–16 months of age) had broad ligament infiltration and increased cervix consistency. The meso-ovarian and uterine ligaments appeared slightly thickened and oedematous. Areas of increased consistency were detected in the cervix, similar to endocervical fold fibrosis, giving the cervix an irregular and frayed appearance. Multiple elastic ovoid formations were identified, arranged in a relatively straight line in the area adjacent to the rectal serosa and perimeter.

**Figure 1:**
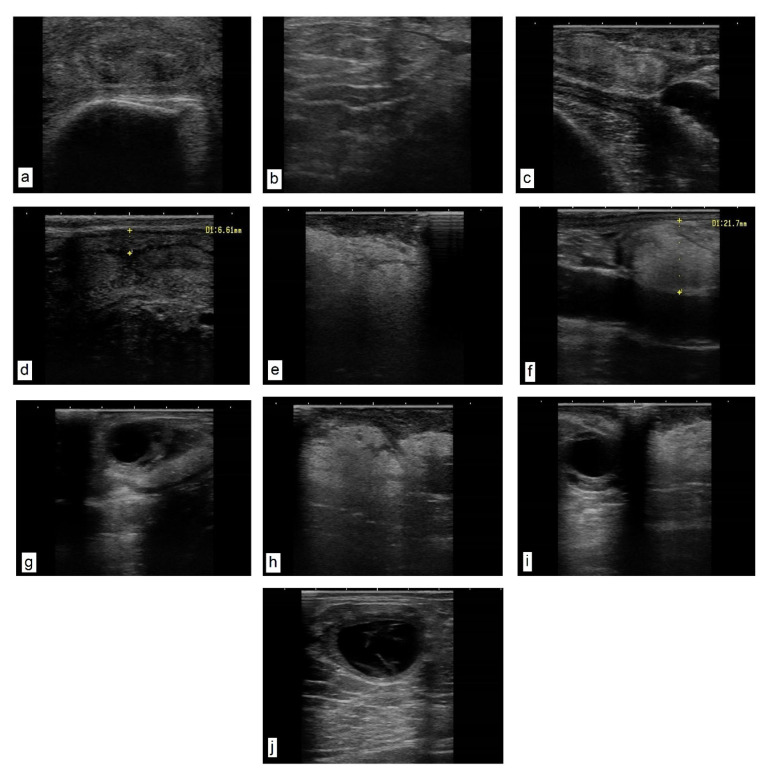
**Ultrasound images of the genital tract.** Transrectal linear probe, 7.5 MHz. (**a**) Heifer cervix, cross-section near the involute flower. The folds of the cervical mucosa have a relatively uniform, denser, hyperechoic texture. (**b**) Cow cervix with proliferation of endocervical folds and their densification. (**c**,**d**) Uterine wall of a cow in puerperium. Normal uterus as an echogenic structure with the presence of a characteristic lumen. (**e**–**i**) Cow in puerperium. (**e**) Hypoechoic (fatty) formation, located paracervically. (**f**) Hypoechoic formation located parasalpingially, adherent to the serosa (longitudinal section) (size 21.7 mm). (**g**) Reactive cow ovary (with dominant follicle). The periovarian area presents a uniform marbled halo with a fatty appearance. (**h**) Sixty days postpartum. Uterine formation, amorphous, homogeneous texture with several areas of densification. (**i**) Reactive ovary with dominant follicle and parasalpingian formation on ligament (meso-ovary), uniform pixelated fatty appearance. (**j**) Heifer ovary 14 days after oestrus. Luteinic ovarian cyst, and a hypoechoic area with septa inside.

A transrectal ultrasound examination of the genital tract in both the heifers and cows in puerperium—in comparison with physiological aspects of the uterus and cervix (Figure 1a,c,d)—showed a diffuse, pathological hyperechogenicity of the cervical folds, suggesting lipid infiltration, as well as the proliferation of endocervical folds (Figure 1b) and the presence of paracervical hyperechoic (fatty) (Figure 1e) and parasalpingian formations (Figure 1f). This explains the change in the topography of the salpinx and the impossibility of oocyte retrieval, as well as the presence of intrauterine (Figure 1h) and paraovarian (Figure 1i) lipogranulomas. In cows in oestrus, the periovarian area shows a uniform marbled halo with a fatty appearance (Figure 1g). In the heifers, 14 days after oestrus, lutein ovarian cysts were found, with a wall thickness of approximately 3 mm and a hypoechoic area with septa inside (Figure 1j).

The haemoleucogram revealed the following: low mean values of erythrocyte haemoglobin concentration (MEHC) in 10/20 cases (50%); monocytosis in 10/20 cattle (50%) and decreased blood platelet count in 8/20 (40%) subjects. Other changes, all synonymous with the association of inflammatory status, revealed a reversal of the leukocyte formula (increase in neutrophils, accompanied by a decrease in lymphocytes) in 4/20 cases (20%), as well as a decrease in the number of eosinophils in 4/20 cases (20%).

Blood biochemical examination revealed a slight increase in AST in 2/20 cases (10%), a slight increase in GGT in 2/20 cases (10%), a slight increase in AST and GGT and mild hypoglycaemia in 2/20 cases (10%) and a slight decrease in serum albumin in 2/20 cases (10%). All of these changes are suggestive of mild liver failure.

**Figure 2:**
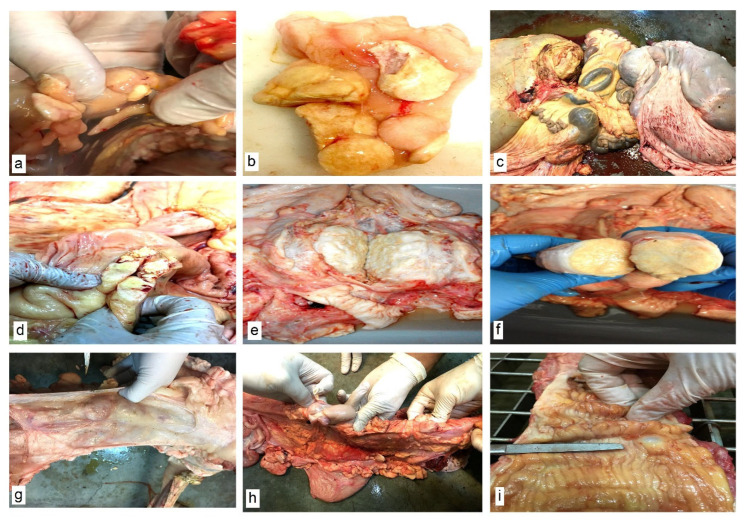
Macroscopic morphological aspects of abdominal organs. (**a**,**b**) Necrosis of abdominal fat. Lipogranulomas. (**c**) Necrosis of mesenteric fat. (**d**,**e**) Lipogranulomas in the broad ligaments of the uterus. (**f**) Paracervical lipogranuloma. (**g**) Lipogranulomas in the uterine horns. (**h**) Parauterine lymph nodes and lipogranulomas in the uterine horns. (**i**) Cervical fibrosis.

*Morphopathological examination results.* In addition to the typical appearance of abdominal lipogranulomas (Figure 2a,b) and mesenteric fat necroses (Figure 2c), numerous lipogranulomas were found in the genital tract of the slaughtered cows in the following areas: in the broad ligaments of the uterus (Figure 2d,e), paracervically (Figure 2f) and in the thickness of the uterine horns (Figure 2g). Fat infiltration of the parauterine lymph nodes (Figure 2h) and fibrosis of the cervix (Figure 2i) were also found.

**Figure 3:**
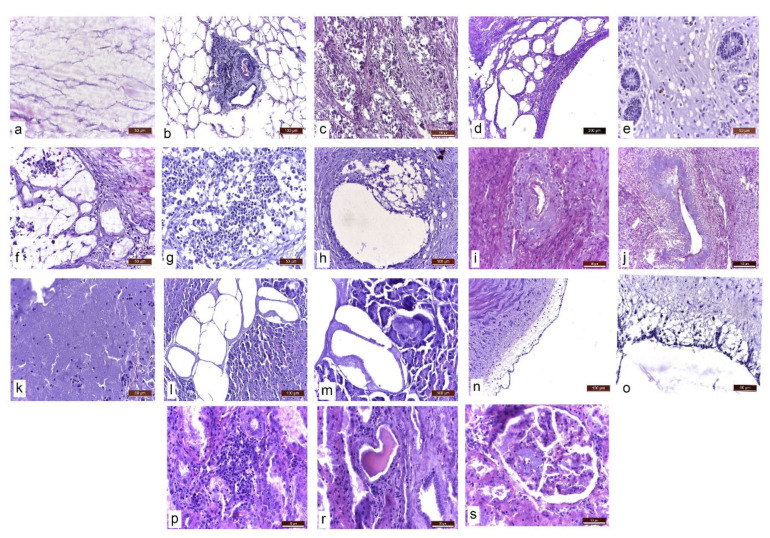
Histopathological aspects of different abdominal tissues and organs. Masson’s trichrome stain. (**a**) Abdominal adipose tissue. Adipocyte necrosis. (**b**) Necrosis and inflammation. Inflammatory reaction with macrophages and giant cells. (**c**) Infiltration of the myometrium with lipids and dissociation of smooth muscle fibres through lipid deposition and associated inflammatory reaction. (**d**) Lipids in the uterine wall. (**e**) Lipids in the chorion of the uterine mucosa. (**f**) Lipids and inflammatory reaction in the uterine ligaments. (**g**) Parauterine lymph node. Infiltration with lipids. Dissociation of lymphoid follicles through lipid deposition. Inflammatory reaction. (**h**) Uterine artery. Lipid deposition in the wall of the uterine artery and secondary inflammatory infiltrate. (**i**,**j**) Amyloidosis of the ovarian artery branches. (**k**) Exocrine pancreas necrosis. (**l**,**m**) Severe lipid infiltration of the pancreas. Lipids are partially saponified. (**n**,**o**) Aorta. Intimal and subintimal lipid infiltration, disrupting the vascular endothelium. (**p**) Kidney. Lymphoplasmacytic interstitial nephritis. (**r**) Hyaline casts in the urinary tubules. (**s**) Amyloidosis of the glomerular capillaries.

Upon histopathological examination, in addition to the typical necrosis and inflammation lesions of the abdominal adipose tissue (Figure 3a,b), abundant lipid infiltrates were found in the internal organs (liver, kidneys) and lymph nodes; an inflammatory reaction was also observed in the thickness of the uterine ligaments. In the uterus, the smooth muscle fibres of the myometrium were dissociated by clusters of macrophages, epithelioid cells and multinucleated giant cells with foamy, vacuolated cytoplasm (containing lipid vacuoles in the cytoplasm) and free lipid deposits (Figure 3c). Abundant lipid infiltration was present in the uterine wall (Figure 3d), the chorion of the uterine mucosa (Figure 3e) and the thickness of the uterine ligaments (Figure 3f). The parauterine lymph nodes showed lipid infiltration, dissociation of lymphoid follicles through lipid deposition and an inflammatory reaction in the presence of macrophages and giant cells (Figure 3g). Lipid deposits and secondary inflammatory infiltrates were present in the muscular uterine artery media (Figure 3h). As a consequence of chronic inflammation and lumen narrowing, amyloidosis of the ovarian artery branches was also found (Figure 3i,j). Lipid vacuoles were even observed in the lumen of capillaries and larger calibre vessels. Extensive necrosis of the exocrine and endocrine pancreas was also observed (Figure 3k). Large masses of partially saponified lipids were identified in the interstitium of the pancreas (Figure 3l,m). The abdominal aorta showed lipid infiltration at the intimal and subintimal levels, which disrupted the vascular endothelium (Figure 3n,o).

At the renal level, as a consequence of the chronic systemic inflammatory process, lesions of lymphoplasmacytic interstitial nephritis (Figure 3p), glomerular capillary amyloidosis (Figure 3s) and altered glomerular capillary permeability—secondary to amyloidosis, resulting in proteinuria (hyaline casts in the urinary tubules) (Figure 3r)—were observed.

**Figure 4:**
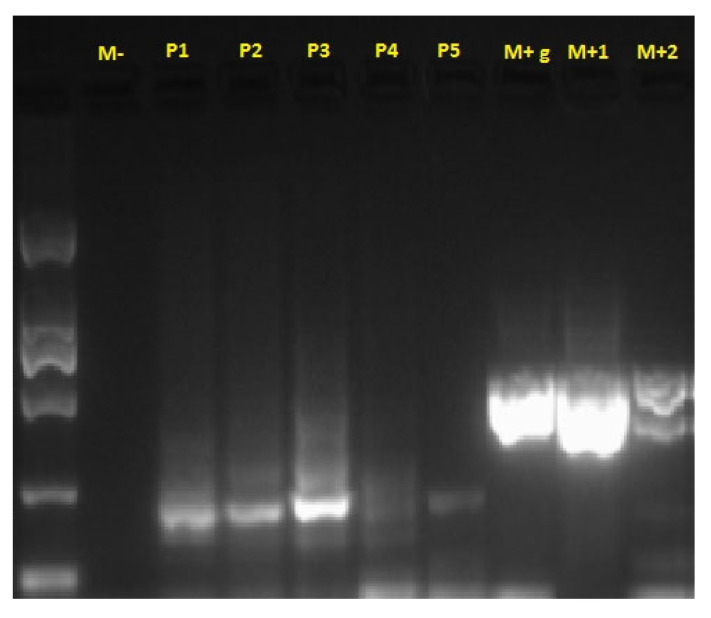
PCR test results for *Mycobacterium* spp. M-: negative control; P1–P5: samples tested; M+g: chicken liver extract control; M+1, M+2: positive controls from *Mycobacterium* spp. culture.

Bacterioscopic and bacteriological examinations, including PCR for *Mycobacterium* spp., were negative (Figure 4). Molecular identification based on the PCR technique has become the gold standard for identifying non-tuberculous mycobacterial species [15].

## 4. Discussions

Generalised steatitis with fat necrosis (‘yellow fat disease’) has been recorded in many species at different ages, being related to dietary vitamin E and selenium deficiency and the excessive intake of unsaturated fatty acids. Associated inflammatory rection is also studied. Immunohistochemical analyses of various fat deposits showed a low incidence of phagocytic immune cell infiltration in cows at the beginning of lactation. The increased accumulation of phagocytic cells in non-pregnant, overconditioned heifers may be related to the larger adipocytes, which secrete higher amounts of chemoattractant adipokines compared to cows at the beginning of lactation [18].

Abdominal fat necrosis in dairy cows is caused by productive forcing (excess fat in the diet), a negative energy balance after calving and a lack of exercise; however, it is possible that the degree of fattening is not very high in order to stimulate significant phagocytic cell infiltration into adipose tissue. Laubenthal et al. [19] investigated the effect of overfeeding on angiogenesis and mitochondrial biogenesis in adipose tissue in non-pregnant and non-lactating cows that received an increasing energy-rich diet for 15 weeks. They found that blood concentrations of oxidative stress markers increased continuously throughout the experimental period, possibly damaging mitochondrial DNA. At the same time, HIF-1α, a major marker of hypoxia, increased, indicating insufficient angiogenesis in rapidly expanding adipose tissue and increasing the production of oxidative stress factors. In the outbreak reported in this study, the significant improvement in disease progression after the introduction of the antioxidant vitamin and microelement supplement supports this hypothesis.

Chronic adipose tissue inflammation in obesity is now a well-established phenomenon, but the pathways initiating the inflammatory cascade are still unknown [20,21,22]. Obese cows, which mobilise more lipids from adipose tissue than thin cows, are prone to developing metabolic disorders (ketosis). This could lead to the increased infiltration of phagocytic immune cells into adipose tissue [23]. It has been hypothesised that neutrophil infiltration into adipose tissue may precede macrophage infiltration as in classical immune responses. It has been shown that shortly (3 to 7 days) after the initiation of a high-fat diet in C57BL/6J mice, neutrophils transiently infiltrate the intra-abdominal adipose tissue parenchyma [21]. Our haematological results, especially monocytosis, observed in 50% of cows, and increased leukocytes, with a reversal of the leukocyte formula in cattle, observed in 40% of subjects, support the hypothesis of chronic inflammation associated with fat necrosis. The ability to regulate inflammation and mitigate postpartum health diseases relies, in part, on the production of inflammatory mediators known as oxylipids. Putman et al. [24] suggest that it may be possible to use oxylipids at early mammary involution to alert dairy producers of cows at risk of disease after calving. Although macroscopic and histological examination showed complex and severe lesions, blood biochemical investigations proved inconclusive, revealing only slight changes suggestive of mild liver failure, an organ with a high capacity for regeneration [2,3].

The necrosis and inflammation of abdominal fat encountered in our study were similar to those described by numerous other authors [2,5,10,21]. Similar pancreatic lesions were reported by Tani et al. [25] and may partially explain the disturbances in Insulin-Like Growing Factor-1 (IGF 1) secretion, involved in glucide and lipid metabolism.

Lipogranuloma deposition in the genital tract in our casuistry partially explains the decreased fertility observed. The main limitations of our study are due to the fact that for economic reasons, we did not investigate hormonal interference with reproductive function. The concentration levels of LH and FSH are disrupted by leptin, a hormone secreted by adipocytes (fat tissue) that either produces poor-quality follicles, prevents ovulation or causes the release of substandard oocytes [26].

Tharwat and Buczinski [14] showed that ultrasonography can be used as an effective noninvasive tool to lead to a diagnosis of potential abdominal fat necrosis in dairy cattle. The combination of rectal gynaecological examination and genital ultrasonography led to the suspicion of fat necrosis in 61.36% of the cows examined. It is worth noting that this is the incidence of cows with clinical symptoms (periuterine deposits identified especially in postpartum cows); the incidence is probably much higher if including asymptomatic ones. Regardless of the clinical expression, the effect of fertility is equally negative.

Ovaries with lipid infiltration have a high degree of endocrine and exocrine dysfunction, with severe repercussions on fertility. Frequently, the oocytes produced by such ovaries are infertile due to lipid infiltration and the biochemistry of the fallopian tubes. If the fallopian tube is not permeable, the gametes do not meet, and fertilisation does not occur. If biochemical changes in physiological secretion occur in the fallopian tube (the presence of lipids in larger quantities and their quality), through the phenomenon of downward migration, the oocyte undergoes a rapid ageing process, becoming covered with layers of mucus and quickly becoming infertile. This ageing process is similar to the ‘snowball’ effect, which increases the diameter and layers of the zona pellucida as the oocyte rolls. In this situation, the spermatozoa are no longer able to penetrate the thickened zona pellucida, and thus fertilisation mechanisms fail [13,26]. In our casuistry, ovarian artery amyloidosis and secondary ischaemia may be additional explanations for infertility.

Pancreatitis is rarely associated with bovine fat necrosis, and it is more common in equines. Affected tissues can be biopsied under ultrasound guidance [2]. This disease must be differentiated from lymphosarcoma, adenocarcinomas and intra-abdominal abscesses. In our casuistry, the multitude and less common location of lipogranulomas raised the question of excluding *Mycobacterium* spp. infections.

In the context of low levels of physical exercise and overfeeding during the production period, farmers make the mistake of adding a higher concentration of lipids to their feed. The effect of these lipids is to compensate for the energy balance and increased milk production, but they cause lipid metabolic imbalances [26]. In recent years, nutritional strategies have emerged as key factors in improving the health status and welfare of animals, as well as in enhancing livestock productivity [27].

## 5. Conclusions

The dramatic decrease in fertility in the cows studied was due to abdominal fat necrosis; this was caused by forced production, which produced severe macro- and microscopic changes in the female genital tract. The deposition of lipogranulomas in the genital tract and its morphological changes partially explain this decreased fertility. Ovarian artery amyloidosis and secondary ischaemia may be additional explanations for infertility caused by poor oocyte quality. Endocrine disorders caused by oxidative stress and multiple inflammatory and degenerative lesions also contribute to infertility. Lipogranulomas can be detected early during routine ultrasound examinations of the genital tract for diagnosing pregnancy.

## Figures and Tables

**Table 1:** Ration composition for lactating cows.

Ingredients	Formula 1 (kg)	Formula 2 (kg)
Corn silage	27	27
Beer slurry	8	8
Corn grain	5.07	5.07
Alfalfa silage	5.0	5.0
Rapeseed meal	3.64	3.64
Wheat	2.6	2.6
Alfalfa hay	1	1
Soybean meal	0.91	0.91
Fat supplement ^1^	0.2	-
Vitamin and trace element supplement ^2^	-	0.2
Calcium carbonate	0.13	0.13
Sodium bicarbonate	0.1	0.1
Salt	0.09	0.09
Total	53.74	53.74
Total DM	24.2	24.01
DM %	44.87	46.43
ME (KJ/kg)	3017.08	2964.76
Crude protein %	16.18	15.18
Fat %	5.08	4.02

DM = dry matter; ME = metabolisable energy. ^1^ For composition, see Table S1. ^2^ For composition, see Table S2.

## Data Availability

The original contributions presented in this study are included in the article/Supplementary Material. Further inquiries can be directed to the corresponding authors.

## Supplementary Materials

The following supporting information can be downloaded at https://www.mdpi.com/article/10.3390/life15081182/s1, Table S1: Composition of fat supplement; Table S2: Composition of mineral-vitaminic premix gived to dairy cattle.

## References

[1] de Bruijn C.M., Veldhuis Kroeze E.J.B., Sloet van Oldruitenborgh-Oosterbaan M.M. (2006). Yellow fat disease in equids. Equine Vet. Educ..

[2] Constable P.D., Hinchcliff K.W., Done S.H., Grunberg W. (2017). Veterinary Medicine. A Textbook of Diseases of Cattle, Horses, Sheep, Pigs, and Goats.

[3] Paul I. (2001). Veterinary Etiomorphopathology.

[4] Neels J.G., Olefsky J.M. (2006). Inflamed fat: What starts the fire?. J. Clin. Investig..

[5] Contreras G.A., Strieder-Barboza C., Raphael W. (2017). Adipose tissue lipolysis and remodeling during the transition period of dairy cows. J. Anim. Sci. Biotechnol..

[6] Smith G.W., Rotstein D.S., Brownie C.F. (2004). Abdominal Fat Necrosis in a Pygmy Goat Associated with Fescue Toxicosis. J. Vet. Diagn. Invest..

[7] Stuedemann J.A., Rumsey T.S., Bond J., Wilkinson S.R., Bush L.P., Williams D.J., Caudle A.B. (1985). Association of blood cholesterol with occurrence of fat necrosis in cows and tall fescue summer toxicosis in steers. Am. J. Vet. Res..

[8] Roche J.R., Friggens N.C., Kay J.K., Fisher M.W., Stafford K.J., Berry D.P. (2009). Body condition score and its association with dairy cow productivity, health, and welfare. J. Dairy Sci..

[9] Zachut M., Contreras G.A. (2022). Mechanistic insights into adipose tissue inflammation and oxidative stress in periparturient dairy cows. J. Dairy Sci..

[10] Caixeta L.S., Omontese B.O. (2021). Monitoring and Improving the Metabolic Health of Dairy Cows during the Transition Period. Animals.

[11] Contreras G.A., Kabara E., Brester J., Neuder L., Kiupel M. (2015). Macrophage infiltration in the omental and subcutaneous adipose tissues of dairy cows with displaced abomasum. J. Dairy Sci..

[12] Jorritsma R., Jorritsma H., Schukken Y.H., Wentink G.H. (2000). Relationships between fatty liver and fertility and some periparturient diseases in commercial Dutch dairy herds. Theriogenology.

[13] Ciornei S. (2024). Reproduction, Reproductive Disorders and Clinical Lecture by Species.

[14] Tharwat M., Buczinski S. (2012). Diagnostic ultrasonography in cattle with abdominal fat necrosis. Can. Vet. J..

[15] Zulfanedi Y., Taniguchi M., Taura Y., Takagi M., Hiyama M., Sasaki N., Tani K., Itamoto K., Nakaichi M., Shigetoshi T. (2019). Bovine Fat Necrosis Finding Around the Stomach with Persistent Vomiting in Japanese Black Cattle. Prosiding Penyidikan Penyakit Hewan Rapat Teknis dan Pertemuan Ilmiah (RATEKPIL) dan Surveilans Kesehatan Hewan Tahun.

[16] Manual of Diagnostic Tests and Vaccines for Terrestrial Animals, Thirteenth Edition 2024. https://www.woah.org/fileadmin/Home/eng/Health_standards/tahm/202406_Chapter_3.01.13_MAMMALIAN%20TB.pdf.

[17] Telenti A., Marchesi F., Balz M., Bally F., Böttger E.C., Bodmer T. (1993). Rapid identification of mycobacteria to the species level by polymerase chain reaction and restriction enzyme analysis. J. Clin. Microbiol..

[18] Akter S.H., Häussler S., Germeroth D., von Soosten D., Dänicke S., Südekum K.-H., Sauerwein H. (2012). Immunohistochemical characterization of phagocytic immune cell infiltration into different adipose tissue depots of dairy cows during early lactation. J. Dairy Sci..

[19] Laubenthal L., Ruda L., Sultana N., Winkler J., Rehage J., Meyer U., Dänicke S., Sauerwein H., Häussler S. (2017). Effect of increasing body condition on oxidative stress and mitochondrial biogenesis in subcutaneous adipose tissue depot of nonlactating dairy cows. J. Dairy Sci..

[20] Elgazar-Carmon V., Rudich A., Hadad N., Levy R. (2008). Neutrophils transiently infiltrate intra-abdominal fat early in the course of high-fat feeding. J. Lipid Res..

[21] Harman-Boehm I., Blüher M.M., Redel H., Sion-Vardy N., Ovadia S., Avinoach E., Shai I., Klöting N., Stumvoll M., Bashan N. (2007). Macrophage infiltration into omental versus subcutaneous fat across different populations: Effect of regional adiposity and the comorbidities of obesity. J. Clin. Endocrinol. Metab..

[22] Heiser A., McCarthy A., Wedlock N., Meier S., Kay J., Walker C., Crookenden M.A., Mitchell M.D., Morgan S., Watkins K. (2015). Grazing Dairy Cows Had Decreased Interferon-Gamma, Tumor Necrosis Factor, and Interleukin-17, and Increased Expression of Interleukin-10 during the First Week after Calving. J. Dairy Sci..

[23] Häussler S., Germeroth D., Laubenthal L., Ruda L.F., Rehage J., Dänicke S., Sauerwein H. (2017). Immunohistochemical localization of the immune cell marker CD68 in bovine adipose tissue: Impact of tissue alterations and excessive fat accumulation in dairy cows. Vet. Immunol. Immunopath..

[24] Putman A.K., Gandy J.C., Contreras G.A., Sordillo L.M. (2022). Oxylipids are associated with higher disease risk in postpartum cows. J. Dairy Sci..

[25] Tani C., Pratakpiriya W., Tani M., Yamauchi T., Hirai T., Yamaguchi R., Ano H., Katamoto H. (2017). Histopathological changes in the pancreas of cattle with abdominal fat necrosis. J. Vet. Med. Sci..

[26] Ciornei S.G., Lopes G., Cenariu M. (2025). Reproductive biotechnologies and challenges in their application. Front. Vet. Sci..

[27] Abbate J.M., Macrì F., Capparucci F., Iaria C., Briguglio G., Cicero L., Salvo A., Arfuso F., Ieni A., Piccione G. (2020). Administration of Protein Hydrolysates from Anchovy (*Engraulis encrasicolus*) Waste for Twelve Weeks Decreases Metabolic Dysfunction-Associated Fatty Liver Disease Severity in ApoE^−^/^−^Mice. Animals.

