# Supplementary material for: Severe Reproductive Disorders After Abdominal Fat Necrosis in Dairy Cattle
Source: Life (Basel). 2025 Jul 25;15(8):1182. doi: 10.3390/life15081182 (PMC12387546; doi:10.3390/life15081182)
Supplement: Supplementary file 1 [file life-15-01182-s001.zip › Table S1. Composition of fat supplement.pdf]

Table S1

Composition of Fat supplement gived to dairy cattle

Parameter	Analytic results
Total fatty matter	99,4%
Moisture	0,4%
Melting point	55°C
Saturated fatty acids	88,7%
