# Supplementary material for: Severe Reproductive Disorders After Abdominal Fat Necrosis in Dairy Cattle
Source: Life (Basel). 2025 Jul 25;15(8):1182. doi: 10.3390/life15081182 (PMC12387546; doi:10.3390/life15081182)
Supplement: Supplementary file 1 [file life-15-01182-s001.zip › Table S2 Composition of mineral-vitaminic premix gived to dairy cattle.pdf]

Table S2

Composition of mineral-vitaminic premix gived to dairy cattle

Mineral supplement based on calcium carbonate, sodium sulphate and magnesium oxide

Composition		UM
Ca	17,3	%
Mg	7	%
**Aditives/Kg**		
**Vitamins and provitamins**		
(3a672a) Vitamin A	1.900.000	U.I.
(3a671) Vitamin D3	575.000	U.I.
(3a700) Vitamin E	13.600	mg.
**Traceelements**		
(3b202) Iodine (calcium iodate)	341,8	mg.
(3b301) Cobalt (Co (II) acetat tetrahidrate)	144,0	mg.
(3b407) Copper (Cu (II) chelated with hydrolysed proteins)	1.440,0	mg.
(3b405) Copper (Cu (II) pentahydrate sulphate)	4.500,0	mg.
(3b505) Manganese (Mn chelated with hydrolysed proteins)	2.160,0	mg.
(3b503) Manganese (Mn sulphate monohidrated)	13.440,0	mg.
(3b612) Zinc (Zn chelated with hydrolysed proteins)	4.800,0	mg.
(3b605) Zinc (Zn sulphate monohidrated)	23.800,0	mg.
(3b810) Selenium (selenizated, inactivated Saccharomyces cerevisiae CNCM I-3060)	48,0	mg.
(3b802) Selenium (sodium selenite)	153,0	mg.
**Probiotics**		
(4a1704) *Saccharomyces cerevisiae* CBS 493.94	19,2	10^9cfu
**Preservative**		
(1a330) Citric acid	18,8	mg.
**Antioxidants**		
(E310) Propyl galate	6,3	mg.
(1b306(i)) Tocopherols extracted from vegetal oils	75	mg.
